# Mapping of Membrane Lipid Order in Root Apex Zones of *Arabidopsis thaliana*

**DOI:** 10.3389/fpls.2015.01151

**Published:** 2015-12-21

**Authors:** Xiaoyu Zhao, Xiran Zhang, Yanli Qu, Ruili Li, František Baluška, Yinglang Wan

**Affiliations:** ^1^College of Biological Sciences and Biotechnology, Beijing Forestry UniversityBeijing, China; ^2^Institute of Cellular and Molecular Botany, University of BonnBonn, Germany

**Keywords:** root apex, Di-4-ANEPPDHQ, membrane order, membrane microdomains, root transition zone

## Abstract

In this study, we used the fluorescence probe, Di-4-ANEPPDHQ, to map the distribution of membrane lipid order in the apical region of *Arabidopsis* roots. The generalized polarization (GP) value of Di-4-ANEPPDHQ-stained roots indicated the highest lipid order in the root transition zone (RTZ). The cortical cells have higher lipid order than the epidermal cells in same regions, while the developing root hairs show very prominent cell polarity with high lipid order in apical region. Moreover, the endosomes had lower lipid order than that of the plasma membrane (PM). Brefeldin A (BFA) treatment decreased the lipid order in both the plasma and endosomal membranes of epidermal cells in the RTZ. The lipid order of BFA-induced compartments became higher than that of the PM after BFA treatment in epidermal cells. Meanwhile, the polarly growing tips of root hairs did not show the same behavior. The lipid order of the PM remained unchanged, with higher values than that of the endosomes. This suggests that the lipid ordering in the PM was affected by recycling of endosomal vesicles in epidermal cells of the root apex transition zone but not in the root hairs of *Arabidopsis*.

## Introduction

According to the classic Fluid Mosaic Model proposed by Singer and Nicolson (1972) in, the plasma membrane (PM) consists of homogeneous lipid bilayers with embedded proteins arranged as mosaic-like structures (Singer and Nicolson, 1972). The lipid bilayer is considered a structural component, providing a hydrophobic barrier to water-soluble substances, while integrated proteins have essential roles in physiological functions. In the original Fluid Mosaic Model, membrane proteins insert into the membranes postulated with uniform lipid bilayers. Almost a decade later, this model was challenged since multiple lines of evidence suggested that the PM consisted by heterogeneously distributed components. Therefore, Karnovsky presented an advanced concept of lipid membrane domains for the first time (Karnovsky et al., 1982; Pike, 2009). Simons and Van Meer (1988) found that apical and basal PM of epithelia have different lipid compostion, the sphingolipid-cholesterol-protein complexes must be delivered to the correct membrane domains from *trans*-Golgi network (Simons and Van Meer, 1988). Later, Simons and Ikonen (1997) proposed a lipid-raft concept and reviewed experimental evidences to link the lipid- raft with signaling processes within cells (Simons and Toomre, 2000). In this so-called lipid-raft model, membrane microdomains are regions enriched with sterol and sphingolipids components, creating a highly ordered and tightly packed region than the surrounding regions enriched with phosphoglycerides. Raft structures provide docking areas for functional proteins, forming sphingolipid-cholesterol-protein complexes that play essential roles in signal transduction and membrane trafficking (Simons and Toomre, 2000). Importantly, the distribution of microdomains showed asymmetric patterns, especially in polarized cells (Simons and Van Meer, 1988; Meer, 1989; Mostov et al., 1992). For example, the polarized distribution of microdomains was observed in nerve cells, polarized T helper lymphocytes, and sperm cells (Tsui-Pierchala et al., 2002; van Gestel et al., 2005a,b; Legembre et al., 2006). However, non-polarized cells, such as lymphocytes and fibroblasts, do not have an obvious polarized distribution of microdomains (Tsui-Pierchala et al., 2002). Obviously, the polar distribution of microdomains in specific cell types has a close relationship with the establishment of cell polarities, which play essential roles in physiological functions in these cells (Martin and Konopka, 2004; Fischer et al., 2008).

The microdomain concept was introduced into plant cytological studies several years later. In Mongrand et al. (2004), purified and analyzed proteins in the detergent resistant membrane (DRM) components, providing the first evidence for the existence of microdomains in plant cells. Since then, various functional proteins associated with the DRM have been identified in different plant species (Morel et al., 2006; Lefebvre et al., 2007; Srivastava et al., 2009). Considering that the DRM fractions do not directly reflect membrane structures (Tanner et al., 2011), further light microscopic and electron microscopic studies were performed to support the existence of microdomains in plant cells. Based on these results, functional proteins and lipid components in microdomains were shown to play different roles (Liu et al., 2009; Raffaele et al., 2009; Furt et al., 2010; Ovečka et al., 2010; Simon-Plas et al., 2011; Li et al., 2012; Malinsky et al., 2013). Polar localization of an auxin eﬄux carrier, the PIN-formed proteins, was shown to have a close relationship with sterols. Willemsen et al. (2003) suggested that sterol methyltransferase 1 (SMT1) is essential in maintaining the polar localization of PIN1 and PIN3. PIN2 polarity via endosomal recycling is also sterol dependent. Polar PIN2 localization on the microdomains is mediated by the auxin ABC transporter ABCB 19 (Men et al., 2008; Titapiwatanakun et al., 2009). The sterol components maintain the polarity of tip growing cells. Liu et al. (2009) found that the pollen tube tips of *Picea meyri* are enriched in sterol microdomains, providing docking domains for NADPH oxidase. Ovečka et al. (2010) revealed that structural sterols are essential in the initiation and tip growing of root hairs of *Arabidopsis*. Polyphosphoinositides are also enriched in DRM fractions, forming signaling microdomains in plant cells (Furt et al., 2010). In non-polarized cells, sterol-enriched components may play a role in the formation of cell plates (Frescatada-Rosa et al., 2014). However, these studies examined the lipid distribution in specific cells. Importantly, an overview of microdomain distribution throughout whole plant organs is not available.

Two fluorescence probes were used in previous studies to visualize the distribution of lipid components in plant cells. Filipin is a sterol-specific dye and di-4-ANEPPDHQ a phase-sensitive fluorescence probe, which can quantitatively image the lipid order in living cells (Boutté et al., 2011). Since the emission peak of di-4-ANEPPDHQ has a blue shift in ordered phase, quantitative measurement of the membrane order can be achieved by ratiometric calculation between images taken from two channels. With this principle, Owen et al. (2011) provided an algorithm for quantitative imaging of lipid order in live cells. The quantified lipid order in the PM reflects the distribution of liquid ordered phase in cells of different tissues or in the different region on the PM (Owen et al., 2010; Kress et al., 2013). Namely, concentrated membrane microdomains can increase lipid order, resulting in the high GP values. In consideration of the relative high cytotoxity of filipin to plant cells (Ovečka et al., 2010; Boutté et al., 2011), we have used di-4-ANEPPDHQ as an optimal probe to visualize and quantify the lipid order in membranes of living plant cells (Zhao et al., 2015). Moreover, di-4-ANEPPDHQ was used for imaging pf polarly growing pollen tubes as well, and it was clearly reported that the apical regions of *Nicotiana tabacum, Picea meyeri* have significant higher GP values than other regions (Liu et al., 2009; Moscatelli et al., 2015).

Our present results provide a map of lipid raft distribution in cells of the root apex zones, indicative of the structural and functional roles of lipid rafts in the determination of cell polarities in root cells. In this map, we show that the root transition zone (RTZ) showed the highest lipid order along the whole root region, the cortical cells showed higher order than those of epidermal cells, and endosomes contained lower lipid order than the PM. Moreover, treatment with brefeldin A (BFA) alters this distribution of lipid order between PM and endosomes.

## Results

### Lipid Order in Root Functional Zones

**FIGURE 1:**
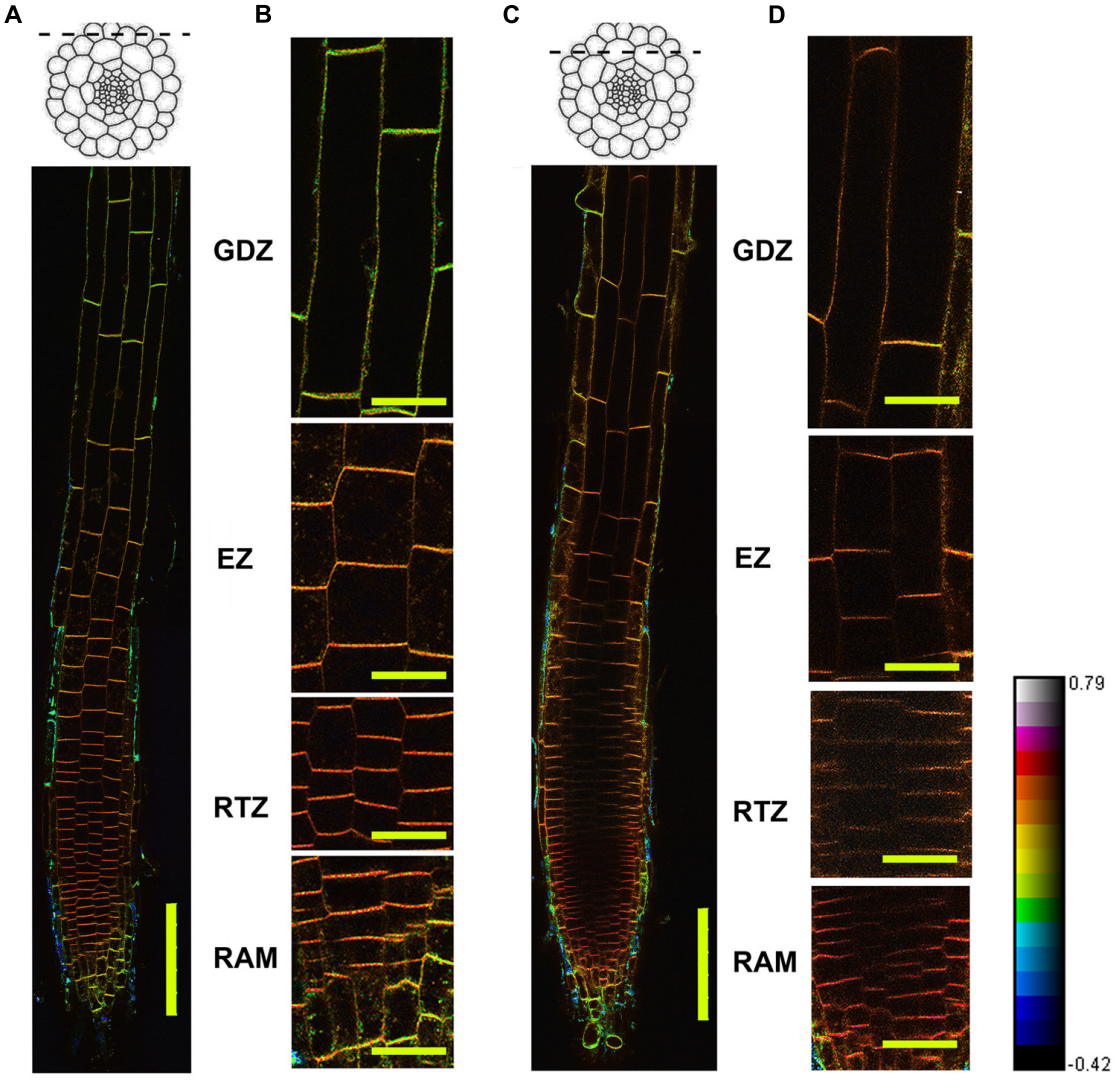
**Distribution of lipid order based on Di-4-ANEPPDHQ staining.** Four-day-old *Arabidopsis* seedlings were stained using Di-4-ANEPPDHQ. Dual-channel images were taken using the confocal laser scanning microscope (CLSM) with the z-series functions. Optical sections from the epidermal and cortical layers were chosen for GP-imaging, the hue, saturation, and brightness (HSB) images were calculated and shown here. **(A,B):** epidermis; **(C,D):** cortex + epidermis. **(C,D)** Are magnified from **(A,B)**. The spectral bar indicates the GP values from the images; purple color represents high GP values and blue color represents low values. Twelve roots were analyzed, all of them showed the same pattern. HSB and GP image of another roots were shown in Supplementary Figure S1. GTZ, growth differentiation zone; EZ, elongation zone; RTZ, root transition zone; RAM, root apical meristem. Bar = 100 μm **(A,B)**; Bar = 25 μm **(C,D)**.

**FIGURE 2:**
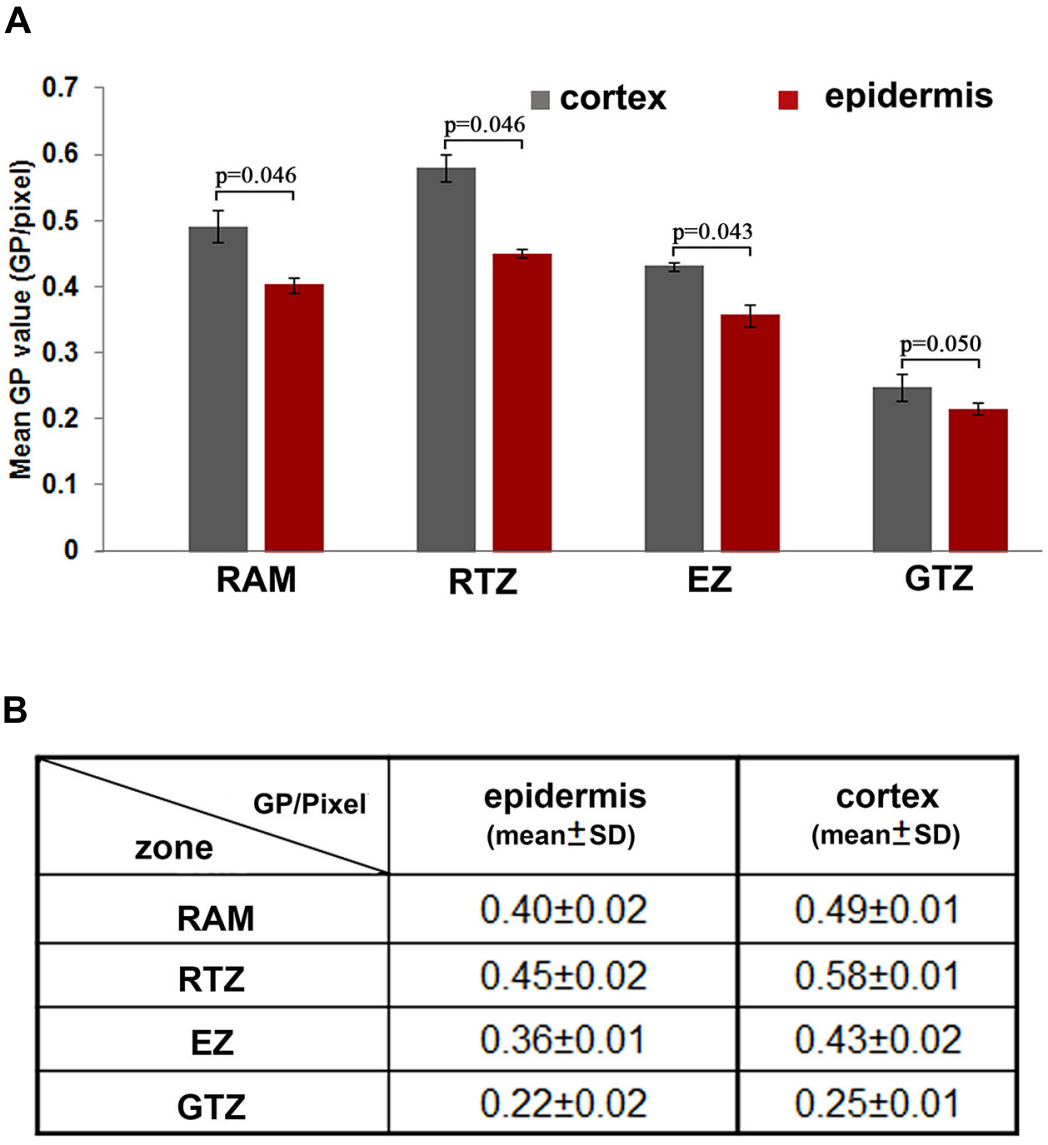
**Distribution of the lipid order along the different cell layers of the root apical region. (A)** Different distribution of GP values in cortical and epidermal cells in root apical functional regions. Blue: GP/pixel measured from the root cortex; red: GP/pixel measured from the epidermis. Statistical significance between the cortical and epidermal layers, according to the Mann–Whitney *u*-test: (*n* = 12, significant difference: *p* < 0.05; no significant difference: *p* > 0.05; *p*-values were presented). **(B)** Mean GP values (GP per pixcel) and standard deviation measured from different regions, the mean values were calculated from 12 roots. RAM, root apical meristem (100 μm from QC); RTZ, root transition zone; 100–250 μm from QC, EZ, elongation zon, 250–550 μm from QC; GTZ, growth differentiation zone, 550–750 μm.

In this study, we used the a method established by Owen et al. (2011) to calculate the GP value of the PM in root apical regions. Pseudo-colored images, named HSB (hue-saturation-brightness) were created by multiplying the GP values by the intensity values in each pixel (**Figure 1**, Supplementary Figure S1). In these images, GP values were indicated by color information, the minimum GP value was set to -0.42 (dark blue) and the maximum value to 0.79 (red to white). The original GP images are shown in supplementary images (Supplementary Figure S1) and the statistical calculation on GP values are presented in **Figure 2**.

From this overview image of the epidermal cells along the whole root apical region, we observed an asymmetric distribution of GP values in different root functional regions (**Figures 1A,B**). From the quiescent center (QC), four functional zones be recognized in root: the root apical meristem (RAM, 100 μm from QC) and root apex transition zone (RTZ, 100–250 μm from QC) regions have obviously higher GP values (red) than those of the elongation zone (EZ, 250–550 μm from QC) and growth termination zone (GTZ) (green to yellow, more than 550 μm from QC). In the overview image of the cortical layer, similar trends of changes in GP values were observed, with the RTZ showing the highest GP value (**Figures 1C,D**). Moreover, the cortical cells showed as a red- and purple-colored boundary, while the epidermal cells in this region were converted into a yellow color, suggesting that the cortical cells have higher lipid order than do the epidermal cells (**Figure 1**).

Since the colorful image provide an intuitive but not precise view on the GP distribution, we further quantified GP values (GP/pixel) in epidermal and cortical cells along whole root apical regions in four distinct functional zones. Among them, cortical cells in the RTZ had the highest GP values (GP = 0.58 ± 0.01) (**Figure 2A**). Behind the RTZ, the GP value continued to decrease until reaching the GTZ, in which cortical and epidermal cells have relative low GP values (GP/pixel < 0.25). Furthermore, when cells differentiated into epidermal and cortical cells behind the meristem, different GP values were clearly represented between these two cell layers (see magnified regions in **Figures 1B,D**). The GP values of the PM in cortical cells were higher (0.58 ± 0.01) than those scored in epidermal cells (0.58 ± 0.02) (**Figures 2A,B**). Similar phenomenon was observed in RAM, RTZ and EZ (**Figure 2**). However, the cortical and epidermal cells from GTZ showed no significant differences according statistic analysis (**Figure 2**).

### Polarity of Lipid Order Distribution in Root Hairs

**FIGURE 3:**
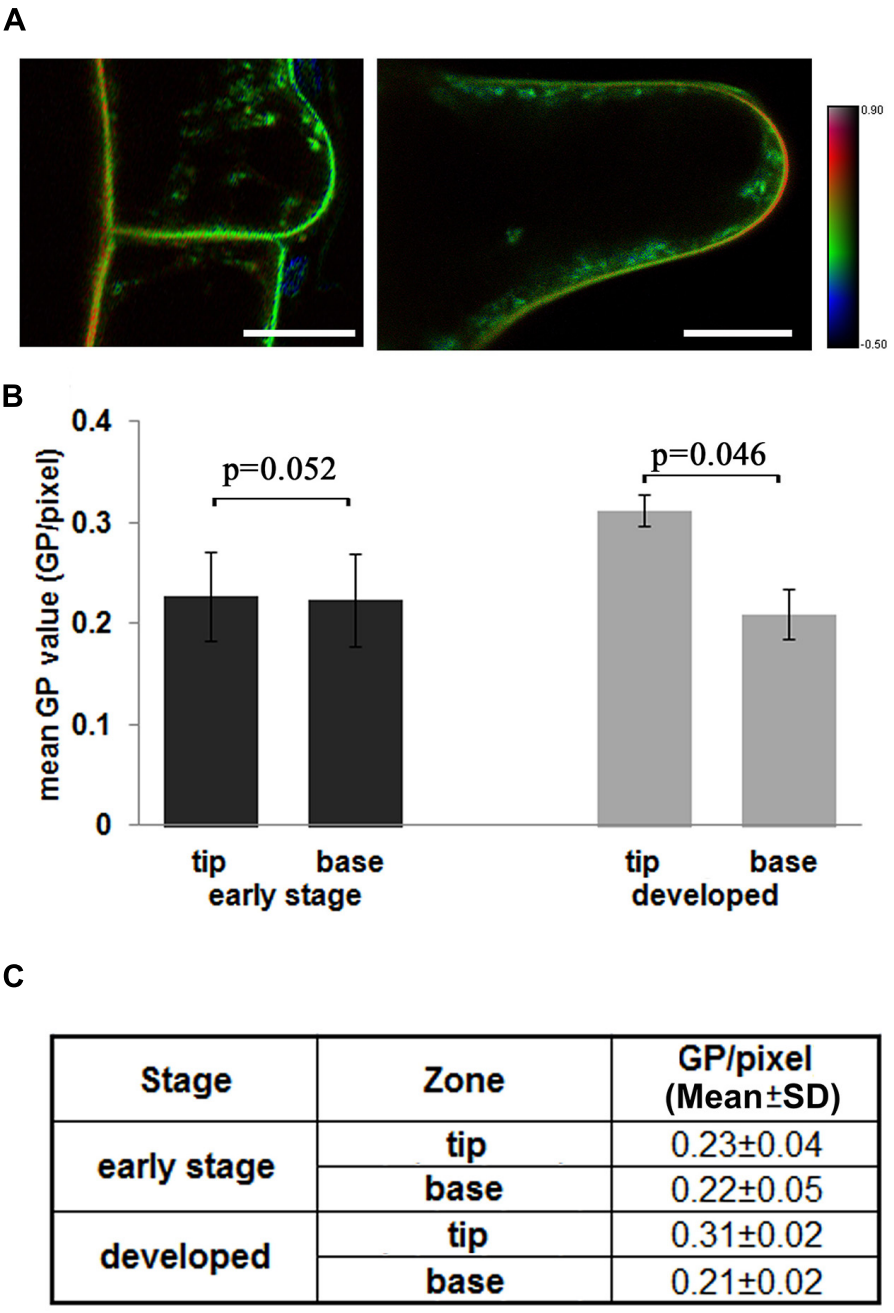
**Distribution of the lipid order in the tip region of growing root hairs. (A)** The 4-day-old *Arabidopsis* seedlings were stained with Di-4-ANEPPDHQ. Dual-channel images were taken using CLSM as longitudinal optical sections, GP images were calculated, and the lipid order were shown in HSB images. Left: a root hair in an early development stage, right: a developing root hair. The color bar indicates the lipid order. The scale bar is 25 μm. **(B)** Different distributions of GP values in early root hairs and developed root hairs. In the initiation stage hairs (<10 μm), whole hair region was measure as tip region, attached epidermal cell was measure as base region. In the tip growing hairs (10–50 μm), the tip region (10 μm from tip) and base region (rest part of hairs) were measured and mean values calculated. **(C)** Mean GP values (GP per pixcel) and standard deviation in different regions. Statistical significance between the cortical and epidermal layers, according to the Mann–Whitney *u*-test. (*n* = 30, significant difference: *p* < 0.05; no significant difference: *p* > 0.05; *p*-values were presented).

We further measured the lipid order in root hairs, which are typical polar growing cells in roots, to understand the distribution of lipid order in polar growing cells. In **Figure 3A**, the polar distribution of the highly ordered PM was not observed during the initiation stage of root hairs (length < 10 μm). The root hair tip region is shown in green, similar to the outer periclinal membranes of epidermal cells (**Figure 3A**). The inner periclinal membranes showed yellow- to orange-colored borders between cortical and epidermal cells, indicating higher membrane orders in this neighbor cortical cell (**Figure 3A**). Meanwhile, the quickly growing root hairs (length between 10 and 50 μm) showed orange- to red-colored apical regions in the HSB image, indicative of a polar distribution of high-ordered membrane domains in the tips of rapidly growing root hair tips. This observation can also be supported based on quantitative measurements (**Figure 3B**). The initiation region of root hairs has similar GP values as those of the attached PM of epidermal cells (GP/pixel = 0.23 ± 0.04 and 0.22 ± 0.04 respectively), while the quickly growing root hairs show significantly increased GP values in the tip region (GP/pixel = 0.31 ± 0.02). The mean GP values and standard deviations were listed in **Figure 3C**, the original GP images of three different root hairs are shown in Supplementary Figure S2.

### Lipid Order of PM and Endosomes

**FIGURE 4:**
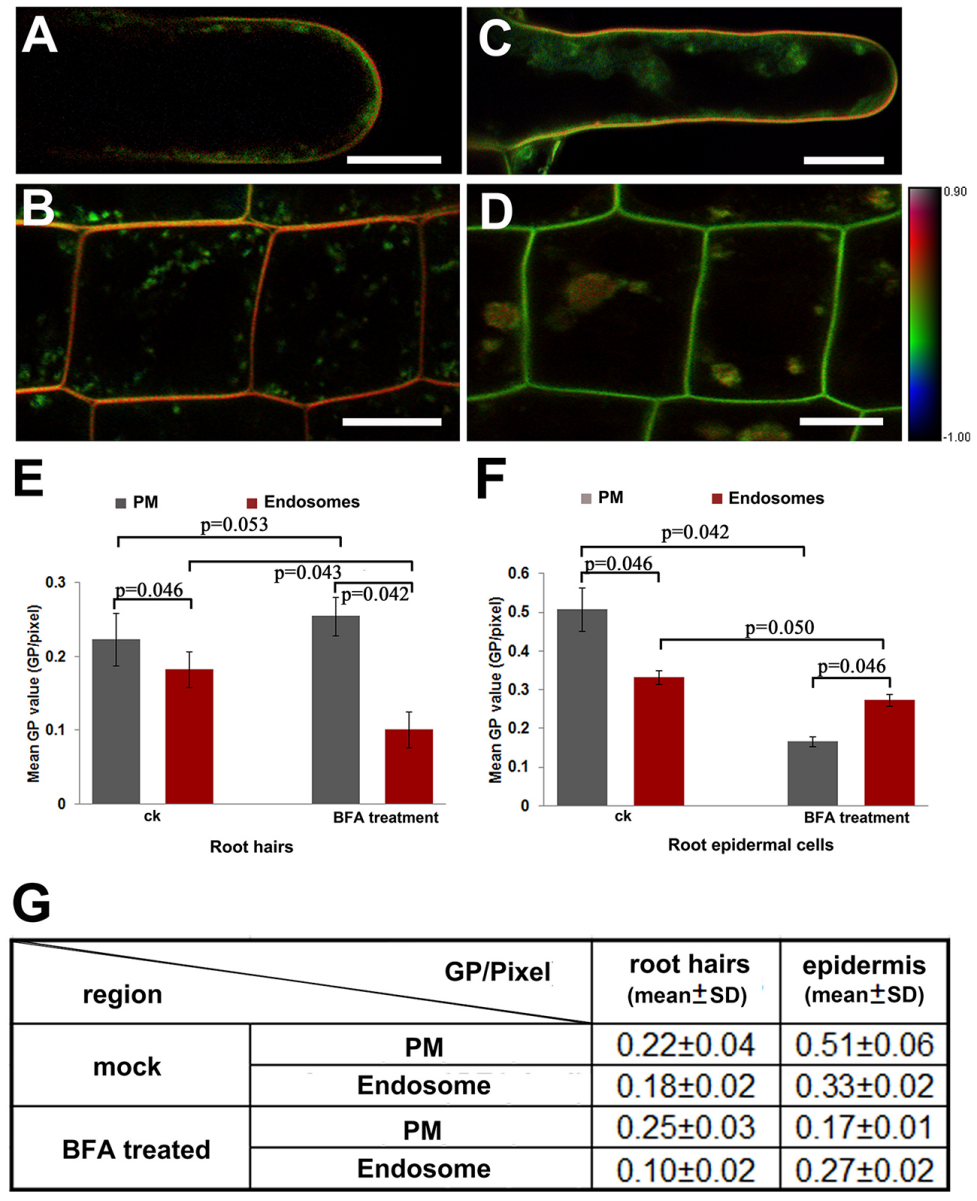
**Asymmetrical distribution of lipid order in the PM and endosomes. (A,B)** Quantitative visualization of lipid order in root hairs **(A)** and epidermal cells in the RTZ **(B)** treated in mock solution (1:1000 DMS for 30 min at room temperature). Scale bar = 25 μm. **(C,D)** Quantitative visualization of lipid order in a root hair **(C)** and epidermal cells **(D)** from an *Arabidopsis* seedling incubated in medium containing BFA (33 μM for 30 min at room temperature). Scale bar = 25 μm. **(E,F)** Distribution of GP values in the PM and endosomes in root hairs **(E)** and epidermal cells **(F)** in mock control (ck) and in BFA sulution. Error bars indicated the standard deviation. Six roots were selected for statistical analysis. Five different RTZ epidermal cells (*n* = 30) and three tip growing root hairs (*n* = 18) in each roots were analyzed. Statistical significance between sample was determined by the Mann–Whitney *u*-test (significant difference: *p* < 0.05; no significant difference: *p* > 0.05; *p*-values were presented). **(G)** Mean GP values and standard deviation between different regions and treatments.

Since endocytosis may be mediated by specific lipid components, we analyzed the Di-4-ANEPPDHQ-stained plant cells to investigate the lipid order of PM and endomembrane components in root cells. The results suggested that the lipid order measured from the PM region were higher than those from the cytosol in both the root hairs and epidermal cells in RTZ (**Figures 4A,B**). After treatment with BFA, an inhibitor of vesicle trafficking and secretion, the endosomal vesicles fused and aggregated into BFA-induced compartments, which were labeled with Di-4-ANEPPDHQ. Meanwhile, the membrane lipid order of measured cells was decreased in both the PM and endosomal compartments of epidermal cells and root hairs. The BFA-treated root hairs remained orange in color in the PM region in the pseudo-color GP images, showing higher lipid order than those in endosomal compartments (**Figure 4C**). The RTZ epidermal cells did not show the same phenomenon after BFA treatment. We found that the BFA-induced compartments were faintly orange; while the PM region becasme green in the pseudo-color GP images (**Figure 4D**). Quantitative analysis confirmed these results (**Figures 4E,F**). In the roots treated in mock control, GP values obtained from PM were always significantly greater than that obtained from cytosol region. The BFA treatment did not change the GP values in PM of root hairs significantly, while that in all other three regions we focused was significantly decreased. Moreover, the BFA-induced endosomal compartments have higher GP values than PM region in RTZ epidermal cells, while root hairs have opposite responses. The mean values and standard deviations were listed in **Figure 4G**. Original GP images of three images from each experiments are shown in Supplementary Figure S3.

## Discussion

Root, the underground part of plants, is not only the anchor of the plant body, but also an active organ in perception, transduction, and reaction to environmental signals, including organic and inorganic factors in the soil. In the growing apex of *Arabidopsis thaliana* primary roots, cells originate in the RAM and pass from the apical meristem to the maturation region. In the *Arabidopsis* root apex, Verbelen et al. (2006) defined four distinct functional zones based on the cellular activities: RAM, RTZ, EZ, and GTZ. After leaving the RAM, cells enter the RTZ, which has a similar size as the RAM. At the basal limit of the RTZ, cells initiate rapid elongation, showing a highly polarized growth pattern along the apical-basal axis (Verbelen et al., 2006). In this study, we revealed that the cortex in RTZ has the highest GP values throughout the root, indicating the PM in this region is highly ordered. This phenomenon might be attributed to the enrichment of various membrane- and cytoskeleton-associated proteins, including auxin transporters, receptors, ion channels, and transporters, as well as NADPH oxidases (Kleine-Vehn and Friml, 2008; Wan et al., 2008; Baluška et al., 2010; Baluška and Mancuso, 2013). Moreover, the RTZ is very active in the flux of auxin, oxygen molecules, nitric oxide, reactive oxygen species, and calcium ions, all showing peak values in the RTZ (Mancuso et al., 2005; Illés et al., 2006; Mancuso et al., 2007; Mugnai et al., 2012). Therefore, it is logic to suppose that the highly ordered membrane domains may provide lipid platforms for protein localization and assembly of protein complexes in signal transduction.

Root hairs are lateral extensions of root epidermal cells characterized by rapid polar growth in GTZ. Here, we found that the rapidly growing root hairs have a peak lipid order at the root hair tips. Importantly, emerging root hairs in the early developmental stages did not show such patterns. This phenomenon is partially in agreement with previous reports. Ovečka et al. (2010) showed that sterol components were concentrated in the root hair tips during both the initiation and tip growing stages. In this early study, filipin-staining methods showed high concentrations of sterol components, a primary component in the construction of lipid microdomains (Ovečka et al., 2010). Moreover, we did not found a clear polar distribution of lipid order in the epidermal and cortical cells in root either. The membrane lipid order can be altered by environmental factors and membrane components, such as the sterol, sphingolipid and functional protein complexes and other environmental factors (Meer, 1989; Mostov et al., 1992; Simons and Toomre, 2000). The GP value method, based on Di-4-ANEPPDHQ staining, scores lipid order quantitatively, but cannot label specific lipid components. This may explain the differences between these two studies. In the future, it is important to verify the contribution of different lipid components to lipid order in small regions of the PM.

Moreover, the RTZ is also very active in endocytosis and endocytic vesicle recycling. Similar to animal and yeast cells, flotilins and remorins were believed to mediate endocytic pathways associated with lipid raft microdomains (Raffaele et al., 2009; Jarsch and Ott, 2011; Li et al., 2012; Bozkurt et al., 2014). Here, we observed changes in lipid order in the endosomal and PM compartments. Quantitative images showed that endosomes have lower GP values than those of the PM in all of the cell types we analyzed, in agree with the early reports (Frescatada-Rosa et al., 2014; Zhao et al., 2015). Frescatada-Rosa used the di-4-ANEPPDHQ to quantify the lipid orders in *Arabidopsis* as well, however, the GP values in this report is much lower than the values we got. In consideration of that the calibration of the G-values may affect the GP values in final calculation, the difference might be caused by different concentration of the dye or the setting of microscopes. To maintain constant GP values along this study, we have calibrated the G-value as 0.5 according to Owen’s protocol (Owen et al., 2011) and have kept all the experimental procedure in identical conditions in our experiments.

Since COPI-coated vesicles were involved in the secretion of sphingomyelin and cholesterol (Brügger et al., 2000), we applied BFA, a powerful inhibitor of secretion in root cells. We found that the total lipid order of the PM and endosomes were significantly decreased in the root epidermal cells after BFA treatment. In plant cells, BFA targets GNOM, a homologue of guanine-nucleotide exchange factors on ADP-ribosylation factor (ARF-GEF), inhibiting vesicular trafficking from endosomes to the PM (Brügger et al., 2000; Richter et al., 2007; Teh and Moore, 2007; Bozkurt et al., 2014). Our results showed treatment with BFA decreased the total lipid order in RTZ epidermal cells and root hairs, implying that the BFA could inhibit PM-endosome trafficking of protein and lipid components, which have roles in maintaining of lipid order. However, more precise experiments are needed in future to investigate the molecular mechanisms of this phenomenon in the different mutant backgrounds relevant for endocytic and exocytic vesicular traffickings.

Interestingly, the polar growing root hairs and epidermal cells in the RTZ showed different distribution patterns of lipid order after BFA treatment. In the RTZ epidermal cells, the lipid order in endosomal vesicles was lower than that in the PM before BFA treatment. BFA reversed this phenomenon when the GP values in the BFA-induced endosomal compartments were higher than those in the PM, even when the GP values in both regions decreased. Since the polar PM of root epidermal cells has highly active endocytosis and endocytic vesicle recycling between the PM and endosomes in the RTZ (Verbelen et al., 2006), inhibition of endosome recycling may enrich ordered PM in recycling vesicles. Thus, the lipid order in the endosomes were higher than those in the PM region. Meanwhile, the polarly growing root hairs did not show changes in PM lipid order after BFA treatment. The GP values of the BFA-compartments scored lower than those of the PM, suggesting they may have involved specific endocytic pathways with different PM components.

## Conclusion

We provided a rough map of zone-specific lipid order in cells of root apical regions of *Arabidopsis thaliana* at the microscopic level. The RTZ showed the highest lipid order along the whole root region, the cortical cells showed higher order than those of epidermal cells, and endosomes contained lower lipid order than the PM. The polar distribution of lipid order was also observed in polarly growing root hairs, but not in non-polarized cells. BFA treatment decreased the total lipid order, showing different effects in different cell types. Considering that the lipid order affected by complicate factors, our results provided elementary clues for investigate microdomain related functions in plant root tissues.

## Materials and Methods

### Plant Materials

We used 4-day-old *Arabidopsis thaliana* (Col 0 ecotype) seedlings in this study. After sterilization with 75% EtOH for 30 s and 1.5% NaClO solution for 1 min, the seeds were washed in sterilized H_2_O (5 × 1 min) and planted in medium containing petri-dishes (½ MS medium with 1% sucrose and 0.4% phytagel). The plates were incubated in culture chambers for 4 days before the experiments (22°C, 12000 lux, light/dark = 16 h/8 h).

### Staining Process and Treatments

Di-4-ANEPPDHQ was purchased from Invitrogen-Life Technologies. The stock solution of di-4-ANEPPDHQ (5 mM in DMSO) was stored in a 200-μl microcentrifuge tube wrapped in aluminum foil at -20°C. *Arabidopsis* seedlings were incubated in staining solution (5 μM Di-4-ANEPPDHQ in ½ MS medium) for 5 min on ice and washed with cold ½ MS for 1 min. Finally, the seedlings were transferred onto a glass slide in 100 μl ½ MS medium for microscopic analysis using a laser confocal scanning microscope (Leica SP5, Germany). BFA was purchased from Sigma-Aldrich^®^. The stock solution of BFA (33 mM in DMSO) was stored in 1 ml microcentrifuge tubes at -20°C. Before treatment, ½ MS medium (1 ml) was added into each tube to get 33 μM working solution. Therefore, 1:1000 DMSO solution was used as mock control.

### Confocal Laser Scanning Microscopic Observations and GP Processing

We used the Leica SP5 confocal laser scanning microscope (CLSM) for the imaging of di-4-ANEPPDHQ-labeled seedlings. A ×63 oil immersion objective (NA = 1.3) was used with an excitation of 488 nm, and the detection ranges of the two channels were set to 500–580 nm and 620–750 nm. Identical microscope settings were maintained for quantitative imaging of membrane components, including the laser power, PMT voltage, and the offset values. After the CLSM imaging process, we followed the early published protocol to generate the GP-images (Owen et al., 2011). The image J macro provided by Owen et al. was downloaded and applied. The GP values were calculated based on the following formula:

(1) $$GP=\left(I_{500-580}-GI_{620-750}\right)/\left(I_{500-580}+GI_{620-750}\right)$$

(2) $$G=\left({GP}_{ref}+{GP}_{ref}{\;\;GP}_{mes}-{GP}_{mes}-1\right)/\left({GP}_{mes}+{GP}_{ref}\,\;\;{GP}_{mes}-{GP}_{ref}-1\right)$$

I_500-580_, I_620-750_: Fluorescence Intensity collected by two channels of CLSM, 500–580 nm and 620–750 nm; G: calibration factor, calculated using formula 2; GP_mes_: measured GP value of the dye in DMSO solution with the constant microscopy setup; GP_ref_: reference value of the dye in DMSO.

To calibrate the G value, we used the G_ref_ = -0.85 as a suggested value (Owen et al., 2011). The G factor was calibrated before each experiment if the microscope setting was changed. To provide an intuitive view, we showed the hue-saturation-brightness (HSB) image in **Figures 1**, **3**, **4**. Since the HSB image represent information of GP values with pixel intensities, we also showed the original GP images in Supplementary Figures S1–S3.

### Quantification of the GP Values in Different Root Apical Regions

Based on the GP images generated by the above described methods, we calculated the different GP value distributions in the root apical regions. Four-day-old *Arabidopsis* seedlings with similar root length were chosen for analysis. We used the ROI manager (Image J/Analyze/Tools/ROI manager) to select the region of interest (ROI) and measured the distribution of pixels in each GP value based on histogram analysis in original GP images (Image J/Analyze/Histogram). From the listed results, pixels with a gray value of 0 were excluded during the calculation of GP values to eliminate regions without fluorescence signals. GP value/pixel was then calculated for each ROI. The G factor was separately calibrated before each run. The Mann–Whitney *u*-test built in the SPSS version 19 was used to determine significance between experiments.

## Author Contributions

XZ and XZ performed the experiments, XZ wrote the manuscript. RL and YQ provided statistical analysis; FB provided important and essential suggestions on designing of experiments and wrote the manuscript. YW designed the experiments and supervised this project. All authors read and approved the final manuscript.

## Conflict of Interest Statement

The authors declare that the research was conducted in the absence of any commercial or financial relationships that could be construed as a potential conflict of interest.

## References

[B1] Baluška F. Mancuso S. (2013). Root apex transition zone as oscillatory zone. *Front. Plant Sci.* 4:354 10.3389/fpls.2013.00354 PMC3788588 24106493

[B2] Baluška F. Mancuso S. Volkmann D. Barlow P. W. (2010). Root apex transition zone: a signalling–response nexus in the root. *Trends Plant Sci.* 15 402–408. 10.1016/j.tplants.2010.04.007 20621671

[B3] Boutté Y. Men S. Grebe M. (2011). Fluorescent in situ visualization of sterols in *Arabidopsis* roots. *Nat. Protoc.* 6 446–456. 10.1038/nprot.2011.323 21412273

[B4] Bozkurt T. O. Richardson A. Dagdas Y. F. Mongrand S. Kamoun S. Raffaele S. (2014). The plant membrane-associated REMORIN1.3 accumulates in discrete perihaustorial domains and enhances susceptibility to *Phytophthora infestans*. *Plant Physiol.* 165 1005–1018. 10.1104/pp.114.235804 24808104 PMC4081318

[B5] Brügger B. Sandhoff R. Wegehingel S. Gorgas K. Malsam J. Helms J. B. (2000). Evidence for segregation of sphingomyelin and cholesterol during formation of COPI-coated vesicles. *J. Cell Biol.* 151 507–518. 10.1083/jcb.151.3.507 11062253 PMC2185577

[B6] Fischer R. Zekert N. Takeshita N. (2008). Polarized growth in fungi-interplay between the cytoskeleton, positional markers and membrane domains. *Mol. Microbiol.* 68 813–826. 10.1111/j.1365-2958.2008.06193.x 18399939

[B7] Frescatada-Rosa M. Stanislas T. Backues S. K. Reichardt I. Men S. Boutté Y. (2014). High lipid order of *Arabidopsis* cell-plate membranes mediated by sterol and DYNAMIN-RELATED PROTEIN1A function. *Plant J.* 80 745–757. 10.1111/tpj.12674 25234576 PMC4280860

[B8] Furt F. König S. Bessoule J. J. Sargueil F. Zallot R. Stanislas T. (2010). Polyphosphoinositides are enriched in plant membrane rafts and form microdomains in the plasma membrane. *Plant Physiol.* 152 2173–2187. 10.1104/pp.109.149823 20181756 PMC2850013

[B9] Illés P. Schlicht M. Pavlovkin J. Lichtscheidl I. Baluska F. Ovecka M. (2006). Aluminium toxicity in plants: internalization of aluminium into cells of the transition zone in *Arabidopsis* root apices related to changes in plasma membrane potential, endosomal behaviour, and nitric oxide production. *J. Exp. Bot.* 57 4201–4213. 10.1093/jxb/erl197 17085753

[B10] Jarsch I. K. Ott T. (2011). Perspectives on remorin proteins, membrane rafts, and their role during plant-microbe interactions. *Mol. Plant Microbe Interact.* 24 7–12. 10.1094/MPMI-07-10-0166 21138374

[B11] Karnovsky M. J. Kleinfeld A. M. Hoover R. L. Klausner R. D. (1982). The concept of lipid domains in membranes. *J. Cell Biol.* 94 1–6. 10.1083/jcb.94.1.1 6889603 PMC2112185

[B12] Kleine-Vehn J. Friml J. (2008). Polar targeting and endocytic recycling in auxin-dependent plant development. *Annu. Rev. Cell Dev. Biol.* 24 447–473. 10.1146/annurev.cellbio.24.110707.175254 18837671

[B13] Kress A. Wang X. Ranchon H. Savatier J. Rigneault H. Ferrand P. (2013). Mapping the local organization of cell membranes using excitation-polarization-resolved confocal fluorescence microscopy. *Biophys. J.* 105 127–136. 10.1016/j.bpj.2013.05.043 23823231 PMC3699755

[B14] Lefebvre B. Furt F. Hartmann M. A. Michaelson L. V. Carde J. P. Sargueil-Boiron F. (2007). Characterization of lipid rafts from *Medicago truncatula* root plasma membranes: a proteomic study reveals the presence of a raft-associated redox system. *Plant Physiol.* 144 402–418. 10.1104/pp.106.094102 17337521 PMC1913791

[B15] Legembre P. Daburon S. Moreau P. Moreau J. F. Taupin J. L. (2006). Cutting edge: modulation of Fas-mediated apoptosis by lipid rafts in T lymphocytes. *J. Immunol.* 176 716–720. 10.4049/jimmunol.176.2.716 16393952

[B16] Li R. Liu P. Wan Y. L. Chen T. Wang Q. L. Mettbach U. (2012). A membrane microdomain-associated protein, *Arabidopsis* Flot1, is involved in a clathrin-independent endocytic pathway and is required for seedling development. *Plant Cell* 24 2105–2122. 10.1105/tpc.112.095695 22589463 PMC3442590

[B17] Liu P. Li R. L. Zhang L. Wang Q. L. Niehaus K. Baluska F. (2009). Lipid microdomain polarization is required for NADPH oxidase-dependent ROS signaling in *Picea meyeri* pollen tube tip growth. *Plant J.* 60 303–313. 10.1111/j.1365-313X.2009.03955.x 19566595

[B18] Malinsky J. Opekarová M. Grossmann G. Tanner W. (2013). Membrane microdomains, rafts, and detergent-resistant membranes in plants and fungi. *Annu. Rev. Plant Biol.* 64 501–529. 10.1146/annurev-arplant-050312-120103 23638827

[B19] Mancuso S. Marras A. M. Mugnai S. Schlicht M. Zársky V. Li G. (2007). Phospholipase Dζ2 drives vesicular secretion of auxin for its polar cell-cell transport in the transition zone of the root apex. *Plant Signal. Behav.* 2 240–244. 10.4161/psb.2.4.4566 19516994 PMC2634134

[B20] Mancuso S. Marras A. M. Volker M. Baluška F. (2005). Non-invasive and continuous recordings of auxin fluxes in intact root apex with a carbon nanotube-modified and self-referencing microelectrode. *Anal. Biochem.* 341 344–351. 10.1016/j.ab.2005.03.054 15907881

[B21] Martin S. W. Konopka J. B. (2004). Lipid raft polarization contributes to hyphal growth in *Candida albicans*. *Eukaryot. Cell* 3 675–684. 10.1128/EC.3.3.675-684.2004 15189988 PMC420133

[B22] Meer G. (1989). Lipid traffic in animal cells. *Annu. Rev. Cell Biol.* 5 247–275. 10.1146/annurev.cb.05.110189.001335 2688705

[B23] Men S. Boutté Y. Ikeda Y. Li X. Palme K. Stierhof Y. D. (2008). Sterol-dependent endocytosis mediates post-cytokinetic acquisition of PIN2 auxin eﬄux carrier polarity. *Nat. Cell Biol.* 10 237–244. 10.1038/ncb1686 18223643

[B24] Mongrand S. Morel J. Laroche J. Claverol S. Carde J. P. Hartmann M. A. (2004). Lipid rafts in higher plant cells purification and characterization of triton X-100-insoluble microdomains from tobacco plasma membrane. *J. Biol. Chem.* 279 36277–36286. 10.1074/jbc.M403440200 15190066

[B25] Morel J. Claverol S. Mongrand S. Furt F. Fromentin J. Bessoule J. J. (2006). Proteomics of plant detergent-resistant membranes. *Mol. Cell. Proteomics* 5 1396–1411. 10.1074/mcp.M600044-MCP200 16648627

[B26] Moscatelli A. Gagliardi A. Maneta-Peyret L. Bini L. Stroppa N. Onelli E. (2015). Characterisation of detergent-insoluble membranes in pollen tubes of *Nicotiana tabacum* (L.). *Biol. Open* 4 378–399. 10.1242/bio.201410249 25701665 PMC4359744

[B27] Mostov K. Apodaca G. Aroeti B. Okamoto C. (1992). Plasma membrane protein sorting in polarized epithelial cells. *J. Cell Biol.* 116 577–583. 10.1083/jcb.116.3.577 1730769 PMC2289323

[B28] Mugnai S. Azzarello E. Baluška F. Mancuso S. (2012). Local root apex hypoxia induces NO-mediated hypoxic acclimation of the entire root. *Plant Cell Physiol.* 53 912–920. 10.1093/pcp/pcs034 22422934

[B29] Ovečka M. Berson T. Beck M. Derksen J. Samaj J. Baluska F. (2010). Structural sterols are involved in both the initiation and tip growth of root hairs in *Arabidopsis thaliana*. *Plant Cell* 22 2999–3019. 10.1105/tpc.109.069880 20841426 PMC2965552

[B30] Owen D. M. Magenau A. Majumdar A. Gaus K. (2010). Imaging membrane lipid order in whole, living vertebrate organisms. *Biophys. J.* 99 7–9. 10.1016/j.bpj.2010.04.022 PMC2895393 20655825

[B31] Owen D. M. Rentero C. Magenau A. Abu-Siniyeh A. (2011). Quantitative imaging of membrane lipid order in cells and organisms. *Nat. Protoc.* 7 24–35. 10.1038/nprot.2011.419 22157973

[B32] Pike L. J. (2009). The challenge of lipid rafts. *J. Lipid Res.* 50 S323–S328. 10.1194/jlr.R800040-JLR200 18955730 PMC2674732

[B33] Raffaele S. Bayer E. Lafarge D. Cluzet S. German Retana S. Boubekeur T. (2009). Remorin, a Solanaceae proteinresident in membrane rafts and plasmodesmata, impairs potato virus X movement. *Plant Cell* 21 1541–1555. 10.1105/tpc.108.064279 19470590 PMC2700541

[B34] Richter S. Geldner N. Schrader J. Wolters H. Stierhof Y. D. Rios G. (2007). Functional diversification of closely related ARF-GEFs in protein secretion and recycling. *Nature* 448 488–492. 10.1038/nature05967 17653190

[B35] Simon-Plas F. Perraki A. Bayer E. Gerbeau-Pissot P. Mongrand S. (2011). An update on plant membrane rafts. *Curr. Opin. Plant Biol.* 14 642–649. 10.1016/j.pbi.2011.08.003 21903451

[B36] Simons K. Ikonen E. (1997). Functional rafts in cell membranes. *Nature* 387 569–572. 10.1038/42408 9177342

[B37] Simons K. Toomre D. (2000). Lipid rafts and signal transduction. *Nat. Rev. Mol. Cell Biol.* 1 31–41. 10.1038/35036205 11413487

[B38] Simons K. Van Meer G. (1988). Lipid sorting in epithelial cells. *Biochemistry* 27 6197–6202. 10.1021/bi00417a001 3064805

[B39] Singer S. J. Nicolson G. L. (1972). The fluid mosaic model of the structure of cell membranes. *Science* 175 720–731. 10.1126/science.175.4023.720 4333397

[B40] Srivastava V. Malm E. Sundqvist G. Bulone V. (2009). Quantitative proteomics reveals a dynamic association of proteins to detergent-resistant membranes upon elicitor signaling in tobacco. *Mol. Cell. Proteomics* 8 2186–2198. 10.1074/mcp.M900090-MCP200 19525550 PMC2742443

[B41] Tanner W. Malinsky J. Opekarová M. (2011). In plant and animal cells, detergent-resistant membranes do not define functional membrane rafts. *Plant Cell* 23 1191–1193. 10.1105/tpc.111.086249 21531862 PMC3101544

[B42] Teh O. K. Moore I. (2007). An ARF-GEF acting at the Golgi and in selective endocytosis in polarized plant cells. *Nature* 448 493–496. 10.1038/nature06023 17653191

[B43] Titapiwatanakun B. Blakeslee J. J. Bandyopadhyay A. Yang H. Mravec J. Sauer M. (2009). ABCB19/PGP19 stabilises PIN1 in membrane microdomains in *Arabidopsis*. *Plant J.* 57 27–44. 10.1111/j.1365-313X.2008.03668.x 18774968

[B44] Tsui-Pierchala B. A. Encinas M. Milbrandt J. Johnson E. M. Jr. (2002). Lipid rafts in neuronal signaling and function. *Trends Neurosci.* 25 412–417. 10.1016/S0166-2236(02)02215-4 12127758

[B45] van Gestel R. A. Brewis I. A. Ashton P. R. Helms J. B. Brouwers J. F. Gadella B. M. (2005a). Capacitation-dependent concentration of lipid rafts in the apical ridge head area of porcine sperm cells. *Mol. Hum. Reprod.* 11 583–590. 10.1093/molehr/gah200 16051681

[B46] van Gestel R. A. Helms J. B. Brouwers J. F. Gadella B. M. (2005b). Effects of methyl-beta-cyclodextrin-mediated cholesterol depletion in porcine sperm compared to somatic cells. *Mol. Reprod. Dev.* 72 386–395. 10.1002/mrd.20351 16044473

[B47] Verbelen J. P. De Cnodder T. Le J. Vissenberg K. Baluška F. (2006). The root apex of *Arabidopsis thaliana* consists of four distinct zones of growth activities: meristematic zone, transition zone, fast elongation zone and growth differentiation zone. *Plant Signal. Behav.* 1 296–304. 10.4161/psb.1.6.3511 19517000 PMC2634244

[B48] Wan Y. L. Eisinger W. Ehrhardt D. Kubitscheck U. Baluska F. Briggs W. (2008). The subcellular localization and blue-light-induced movement of phototropin 1 – GFP in etiolated seedlings of *Arabidopsis thaliana*. *Mol. Plant* 1 103–117. 10.1093/mp/ssm011 20031918

[B49] Willemsen V. Friml J. Grebe M. Van den Toorn A. Palme K. Scheres B. (2003). Cell polarity and PIN protein positioning in *Arabidopsis* require STEROL METHYLTRANSFERASE1 function. *Plant Cell* 15 612–625. 10.1105/tpc.008433 12615936 PMC150017

[B50] Zhao X. Li R. Lu C. Baluška F. Wan Y. (2015). Di-4-ANEPPDHQ, a fluorescent probe for the visualisation of membrane microdomains in living *Arabidopsis thaliana* cells. *Plant Physiol. Biochem.* 87 53–60. 10.1016/j.plaphy.2014.12.015 25549979

