# Supplementary material for: Mapping of Membrane Lipid Order in Root Apex Zones of Arabidopsis thaliana
Source: Front Plant Sci. 2015 Dec 21;6:1151. doi: 10.3389/fpls.2015.01151 (PMC4685293; doi:10.3389/fpls.2015.01151)
Supplement: Supplementary file 1 [file Data_Sheet_1.PDF]

Supplementary Data

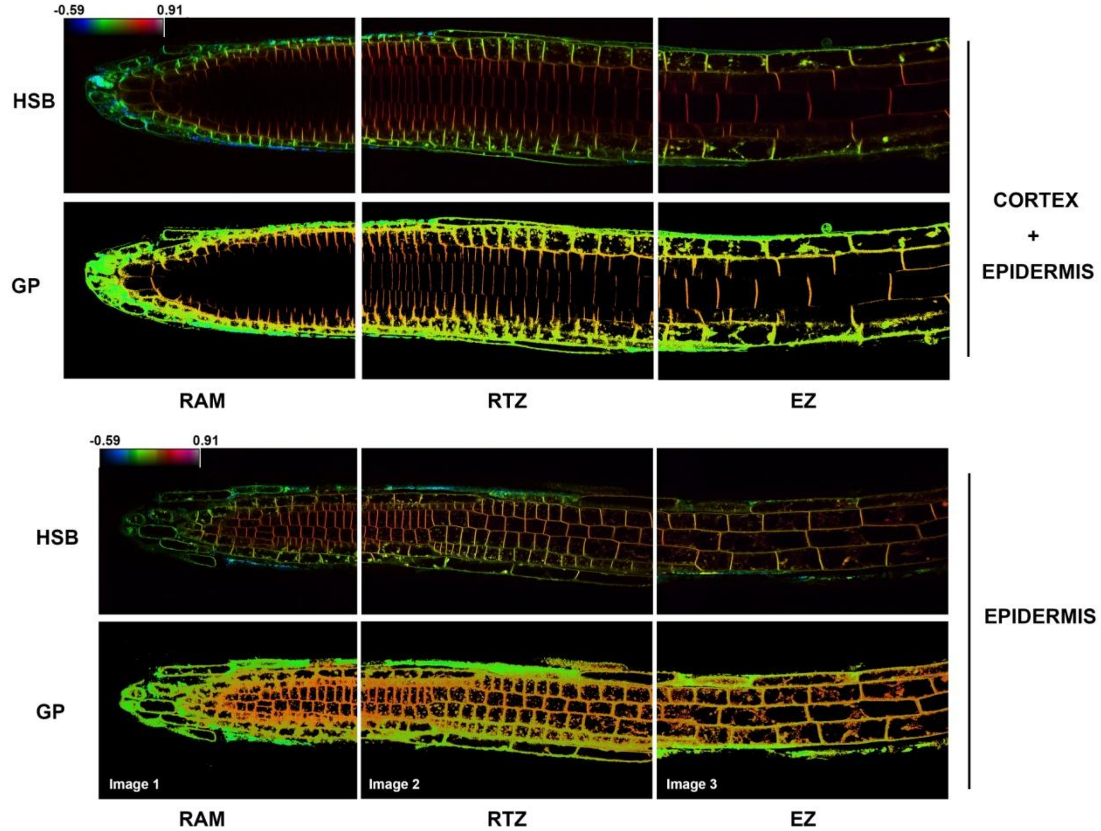

Figure S1: comparison of GP image and HSB image of di-4-ANEPPDHQ-labeled root tip region of Arabidopsis.

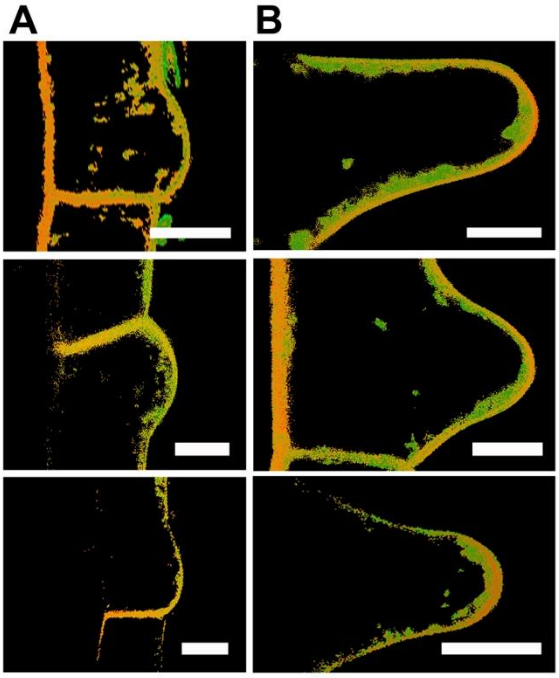

Figure S2: GP images of initiating and elongating root hairs.

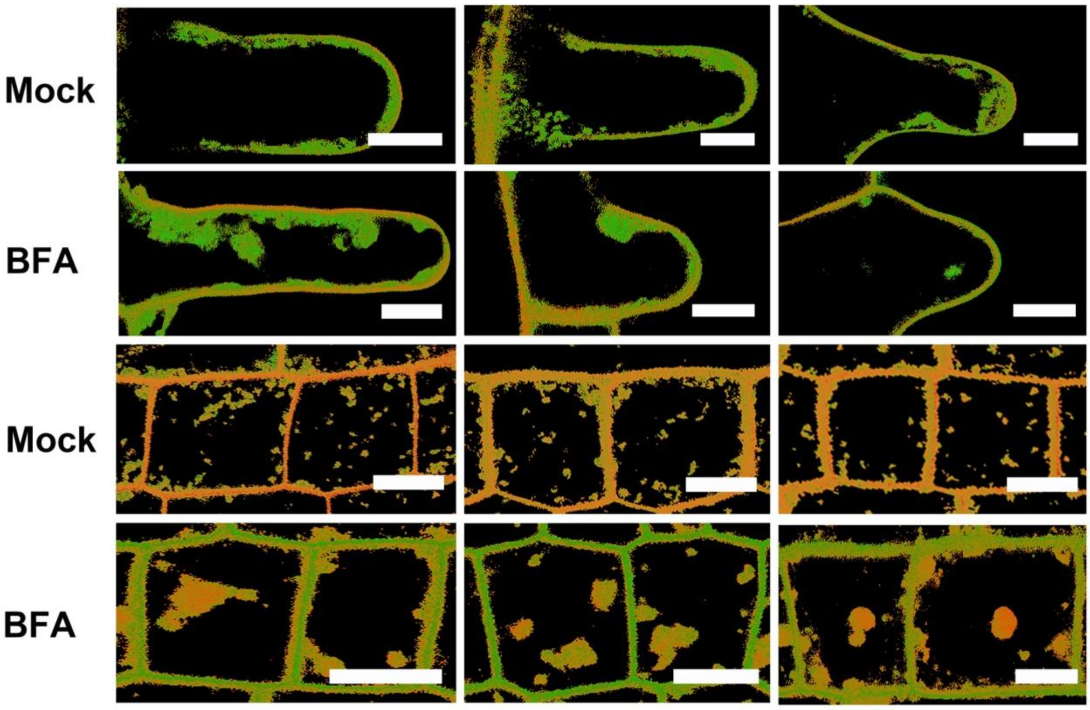

Figure S3. GP images of root hairs and epidermal cells in Arabidopsis root with/without BFA treatment.
